# Effects of low-powered RF sweep between 0.01-20 GHz on female *Aedes Aegypti* mosquitoes: A collective behaviour analysis

**DOI:** 10.1371/journal.pone.0178766

**Published:** 2017-06-05

**Authors:** Abdul Halim Poh, Mahmoud Moghavvemi, M. M. Shafiei, C. S. Leong, Yee Ling Lau, Faisal Rafiq Mahamd Adikan, Majid Bakhtiari, Mahmood Ameen Abdulla Hassan

**Affiliations:** 1 Department of Electrical Engineering, Faculty of Engineering, University of Malaya, Kuala Lumpur, Malaysia; 2 Centre of Research in Applied Electronics, Faculty of Engineering, University of Malaya, Kuala Lumpur, Malaysia; 3 University of Science and Culture, Tehran, Iran; 4 Department of Parasitology, Faculty of Medicine, University of Malaya, Kuala Lumpur, Malaysia; 5 Faculty of Computing, University Technology Malaysia, Johor, Malaysia; 6 Department of Biomedical Science, Faculty of Medicine, University of Malaya, Kuala Lumpur, Malaysia; National Taiwan Ocean University, TAIWAN

## Abstract

There are many products claiming to be an electronic solution towards repelling mosquitoes. Several reviews were published in debunking these claims. However, there is a lack of a systematic study on effects of electromagnetic (EM) or more specifically, radio frequency (RF) waves against mosquitoes due to the conclusions made in those years. Therefore, we attempt to establish a fundamental study on female *Aedes Aegypti* (Linnaeus) mosquitoes by quantifying the collective behavior of the mosquitoes against a continuous stream of low-powered RF signals via a broadband horn antenna using image processing methods. By examining the average lateral and vertical positions of the mosquitoes versus frequency and time, the data shows negligible consistency in the reactions of the mosquitoes toward the different frequencies ranging from 10 to 20,000.00 MHz, with a step of 10 MHz. This was done by examining 33 hours of spatiotemporal data, which was divided into three sessions. All three sessions showed totally different convolutions in the positions in arbitrary units based on the raster scan of the image processing output. Several frequencies apparently showed up to 0.2–70% shift in both lateral and vertical components along the spectrum, without repeatability for all three sessions. This study contributes to the following: A pilot study for establishing the collective effects of RF against mosquitoes, open-source use, and finally a low-cost and easily adaptable platform for the study of EM effects against any insects.

## I. Introduction

A detailed understanding of the issues around mosquito control is crucial as current methods are still being put into question for further improvements [1]. Electronic mosquito repellents, as one of the supposed vector controls are mostly ultrasonic types, and have made way into the consumer electronics for quite some time although reviews have been made on the inefficacies of the products [2]. However, there is a huge gap in the conclusions made with several available publications, where only a few selected frequencies between 125–74,600 Hz was reported and the details on the electronic signals are ignored in most reports, i.e the type of waves, whether they were radio frequencies (RF), optical, or acoustic signals, the kind of antenna used if the signals were electromagnetic waves, the circuitry used, and so on. Therefore an attempt to systematically verify this assertion was made, and to establish a more detailed data-based approach for reporting purposes.

The biological effects of RF have been studied for quite some time. An unwritten law in the industry of RF technology was the assertion that all RF is safe provided they were kept below the limits, and on the whole, comprehensive standards have been established for EMF radiation in the non-optic region, from 0 to 300 GHz. The World Health Organization (WHO) and International Commission on Nonionizing Radiation Protection (ICNIRP), among others, are bodies which monitor the radiation safety policies in the industry, although several other bodies have differing policies such as the Eastern European (EE) standards [3]. ICNIRP has defined limits to the electric field levels (V/m) for varying frequencies, allowing higher power levels up to 100 kHz and lower toward 100 GHz, whereas IEEE delved further into the magnetic and electric field thresholds levels with emphasis on the 2.4 GHz region, which corresponds with conventional cell phone frequencies [3].

From 0 to 10^22^ Hz, potential effects of electromagnetic radiation have been generally divided into non-ionizing and ionizing types, with four general characteristics, namely the Extremely Low Frequencies (ELF) and Very Low Frequencies (VLF), which has non-thermal effects, and up to the microwave region, which has thermal effects, and infrared (IR) and ultraviolet (UV) which has photoelectric effects, and finally ionizing waves such as the X-Ray and gamma rays, which are hazardous [4].

Reviews have compiled effects of high-powered electromagnetic field effects in Extremely Low Frequencies (ELF), defined as ranging less than 100Hz, on several insect types including Drosophila and bees [5]. The effects range from cellular mitotic activity of cells to animal fatalities, with nuances to different insect types. Behavioral changes were also reported on several insect types, from drinking habits to interspecies killings. However, how these waves specifically affect the insects are not resolved. Adverse effects have also been reported on personnel working in high-voltage substations, said to affect their central nervous system and cardiovascular activity. However, none were reported for mosquitoes [5]. It is likely that the use of ELF for mosquitoes is unsafe even if any form of vector control can be observed.

As we go along the spectrum, dosimetric studies of microwave radiation, which range from very low frequencies up to 300 GHz, have demonstrated some concern, although not in an imminent manner [3]. Effects of cell phone RF radiation with frequency of 850 MHz on human semen have been observed in terms of oxidative effects [6]. Effects have been observed in the animals tracked using radio transmitters operating between 27–401 MHz, including oxidative damage, behavioral and genotoxic effects, fertility and navigational disruption [7]. Telecommunication antenna radiation operating between 800–2,600 MHz have been known to affect several insect species, included developmental delays and reproduction problems in fruit flies (*Drosophila melanogaster*), colony strength degradation, navigation interruption in honeybees and cockroaches which leads to straying, and disruption of the olfactory faculties in ants [8]. Although most reports are made in-lab, these recent findings prompted the call to investigate further the effects of commercial RF exposure on wildlife [9], which was answered in a recent study by comparing the abundance of pollinators in RF and non-RF radiated areas. For the first time, it has been shown that RF indeed affects the pollinators [8].

Although reputedly safe, microwave ovens commonly use 2.4 GHz albeit on a much higher power level, as industrial grades may rate up to 1800 W, which reportedly interfere with the conventional communication frequencies, which operates on the same frequency [10]. Terahertz (THz) frequencies were also reviewed, including the use against fruit flies (*Drosopholia melanogaster*), where effects included gene expression at 6.69 THz. Also, the use of IR was reported with somatic mutations observed, at 3.69 THz [11]. However, none of the frequencies ranging from Low Frequencies (LF) up to microwave have been used towards mosquitoes in literature.

Although a call to explain the biological effects of RF in individual research was made due to the numerous factors contributing to the peculiarities, there is a consensus on the thermal effects it induces [12]. Certain RF frequencies (and at a certain power level) is understood as a heating mechanism due to the oscillations of polarized molecules and charged ions while interacting with RF, which recent applications still find its way in postharvest control for disinfecting insect pests [13, 14]. To date, the biological effects of low-powered RF, for instance emitted by cell phones towards humans have been inconclusive [4, 6, 15]. However, a review has highlighted the possibility of cellular interactions with RF, including cell plasma membrane, genome, and the presence of water, which may the cause of the diversions from normal activities of various bacteria strains. This was articulated in the highlights of previous research, which observed altered ionic transport processes and enzymatic activity in the plasma membranes of bacteria when exposed to certain frequencies between 50–73 GHz [16]. The navigation of honeybees were said to be affected due to the magnetite structures in the bee, acting as a natural compass interfered by telecommunication antenna radiations [8].

Based on the previous studies, we would like to define several criterion on studying the effects of RF on mosquitoes, including:

1. Including electromagnetic frequencies excluding the ionizing regions which are unsafe,
2. RF Power levels kept within the thresholds of the governing bodies which conforms to local standards (WHO, ICNIRP, IEEE, and others), and
3. Performed by quantifying the mosquitoes’ activities, either by tracking or swarm behavior within an enclosure

Based on these criterion mentioned above, the materials and methods to fulfill them will be discussed in the following section.

## II. Materials and methods

In this section, there are three main components which we employ in this study. The first will be the chamber design used to house the mosquito swarms, where rationale, dimensions, components and materials used for the construction will be elaborated. The second will be the RF setup, which includes the function generator including methods of frequency management, the amplifiers, and the antenna chosen for the scheme. The third and final component will be the quantification method, which uses a custom-made software to map the two-dimensional mosquito positions in the chamber and to study the collective behaviour by averaging techniques.

### A. Chamber setup

**Fig 1:**
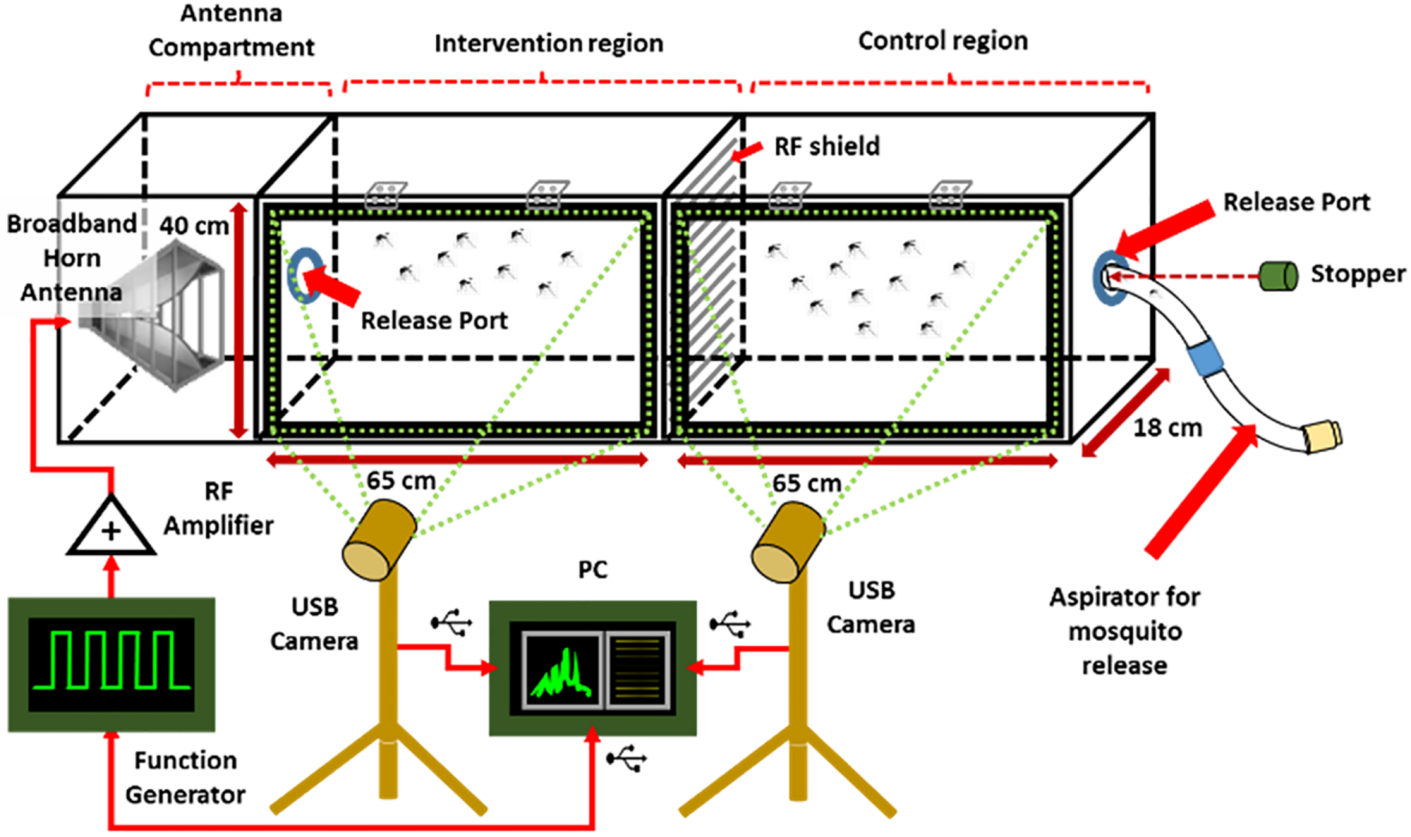
Evaluation chamber.

There are various designs for analysing insects in-lab with various justifications [17–19]. However, in this study, a more specific design is required as shown in Fig 1, which is a modified version from a previous closely related research [20].

The chamber design is custom made with stacked plywood mounted using plastic brackets (dimensions 176.0 x 47.0 x 18.0 cm) enclosed with a transparent acrylic panel to access visuals on the subjects. The whole chamber is painted white, for contrasting of the mosquitoes and the background for digital quantification. The acrylic panel is mounted using hinges, which allows opening the chamber for cleaning inside the compartments of any residue from the experiments, since fungi may grow on the mosquitoes’ faeces and affect the experiment. Three compartments are introduced, namely the antenna compartment for placing the antenna, an intervention compartment for testing of the radio frequencies against the mosquitoes, and a control compartment.

The control and intervention compartments are separated from each other by an RF shield to absorb or dispel any RF radiation towards the mosquitoes. Both compartments, including the gaps around the release ports are completely sealed with general purpose silicone sealant and reused elastomeric nitrile foamed rubber (Superlon^™^) for extra precaution. Airways for breathing are provided by allowing small gaps between the acrylic panel and the foamed rubber. The chamber area is provided with diffuse lighting for the imaging process to occur satisfactorily.

### B. Antenna and detection setup

Different antennas are used for different applications with different properties, which primarily depend on the frequency range and output power. In previous studies for biological effects of RF, starting from ELF, long cable networks (part of the now ceased Project Sanguine [21]) as mentioned in a study on the biological effects of ELF were used [5, 8]. Omni-directional antennas were used for the study of effects of RF against wildlife tracking [7] and human semen [4, 6, 15], and base station antennas against pollinators [8]. In this study, a broadband horn antenna is chosen (LB7180, Ainfo^™^). The antenna has a frequency range of 0.7-20GHz, 12dB typical gain, linear polarization, and weighing around 1.5 kg. The antenna is used to deliver the generated RF wave to the subject of the study. The large bandwidth of the antenna provides enough flexibility for us to use a single antenna for variety of frequencies. The antenna was mounted into the designated compartment as shown in Fig 1 and oriented in the direction toward the intervention region directly.

Referring to Fig 1, a wideband function generator, capable of generating from 0.01–20 GHz (HMC-T2100, Hittite Microwave Corporation^™^) is used as the source of RF wave for this study. This signal generator is connected to a super-ultra-wideband amplifier (ZVA-213+, Mini Circuits^™^) using a both male SMA-to-SMA coaxial cable. A constant DC voltage of 12V supplies the amplifier 400 mA using a standard adjustable DC power supply. The amplifier is connected to the antenna using a both male SMA-to-N coaxial cable to the amplifier. There is a setup tradeoff which includes the use of frequencies lower than 0.7MHz for the broadband antenna, and the amplifier which operates from 0.8MHz. However, there is no evidence of a cutoff beyond the operating frequencies for both devices, and therefore with only 3% outlier beyond the operating frequencies, we choose to include the frequencies beyond the operating parameters for wider coverage.

Two units of off-the-shelf USB-connected web cameras are used for the mosquito motion recording (Sirius USB2.0 and Logitech S7500). The cameras are mounted on standard aluminum camera tripods and placed in front of the panels of the chamber. Pre-test alignment was performed by activating video mode and adjusting the best 3D orientation in order to allow the whole view of the respective compartments to come into view. In order to use these cameras for data collection, video applications are not required for installation, since the default driver installation upon USB connection is sufficient for the OpenCV^™^ software to operate the captured feed.

### C. Mosquito preparation & release

Mosquito preparation was carried out similar to our previous work [20]. For each testing sessions, approximately 150–250 female *Aedes aegypti* (Linnaeus) mosquitoes aging between 5–9 days were collected from Department of Parasitology, University of Malaya. The mosquitoes were colonized under controlled insectary settings with humidity levels of 75–90%, temperature 27±2°C, and equal light and dark cycles per a 24-hour day. The mosquitoes were nourished with a blend of powdered milk, cat fodder, bovine liver and mice chow with weight ratio of 1:1:1:2. Following the hatching, the adults were sustained with 10% sucrose solution. The mosquitoes are then mouth-exhaled into smaller containers, supplied with 10% sucrose solution wetted cotton and transported to the test chamber as shown in Fig 1. No blood meals were provided before the testing. The reason female *Aedes aegypti* mosquitoes were used was due to the fact that only females are known to take blood meals and thus turn into disease vectors, hence the importance of understanding their response against RF [22]. Also, blood meals were excluded to optimize the host-seeking behavior of the mosquitoes [23].

After cleaning the chamber from contaminants from mosquito residue and dust, the mosquitoes are then mouth-exhaled into the test chamber, and a stopper is immediately applied to both compartment openings. This is performed for both compartments, where each housed approximately 100–150 mosquitoes. The mosquitoes are then allowed to settle down for half an hour.

### D. Software setup, data collection and treatment

Prior to data collection, there are three core software which requires initialization, namely a custom made software using OpenCV^™^ v3.1 coded within Visual Studio Community^™^ 2013 (VS2013^™^), and finally HMC-T2200 (Hittite Microwave Corporation^™^), which operates the function generator via a USB connection, and allows for automatic adjustments of a plethora of parameters including the frequency, delay times, signal power, and others. All software used in this study is open-source or free with plenty of tutorials for setup.

The mosquito behavior quantification is performed using OpenCV^™^ by utilizing Suzuki’s method of topological structure extraction analysis by border following [24] algorithm, a function readily made available for use in the coding library, which was used in our previous research for quantifying aggregate mosquito positions in 2D [20]. Similar to the previous work, the image processing library allows for mapping the position of the mosquitoes behind the background, excluding the ones resting in the edges of the chamber, as also implemented in a previous study on mosquito flights against human odor [25]. The data output returns collective *x-* and *y-* coordinates as a function of response of the mosquitoes against the frequencies which will be radiated in the direction of the swarm. Similar to our previous study, the coordinates’ origin start from the top left of the image matrix. We simplify the quantification collectively by averaging any 2D motion registered in the system since previous studies have established the relevancy of 2D mosquito flight quantification [26].

Upon the release of the mosquitoes, the quantification starts initialization from executing the OpenCV^™^ application through VS2013^™^ application (.exe) file, or via debugging the program in VS2013^™^ coding window tab. A boundary is defined within the raster shown in the main feed of the camera by adjusting the trackbars which correspond to the position and the size of the boundary in 2D. This will simplify the captured blobs in the video feed and registers a data collection with less noise **(See** S1 Video**)**. The data output will appear according to the path input during the startup of the application in.txt files upon sliding item “Start” from 0 to 1. This code development is performed for one camera, therefore for the other camera another debugging file must be used with modifications to the code (**See** S1 Source code and files). Given the difficulty in maintaining precision on the detection of mosquitoes using machine vision, a basic noise level is expected from the data gathering, where lighting, camera resolution and placement plays a critical role in minimizing the errors. However, detected mosquitoes within the image feed will introduce the displacement in the spectrum of the detection, where the shifts will indicate a response. Initially before proceeding toward the testing, a complete run on the experimental setup with all the hardware in place without powering on the devices is performed by collecting data for approximately 40–80 minutes to observe the response, designated as the null test. The data output is collected for comparative purposes.

Next, the application HMC-T2200 (Hittite Microwave Corporation^™^) to control the frequency of the signal into the amplifier and the antenna is initialized. For the periodic sweep test, the starting frequency is fixed from 10 MHz, with an interval of 20 seconds, frequency step of 10 MHz and a fixed power output of 10 dBm in continuous sweep mode, whereas the input power of the amplifier is fixed to a maximum 4.8W (as per typical settings by fixing 12V and 400 mA in the ZVA 213+ amplifier datasheet). In this test, power levels from the source are kept constant due to limits of the power amplifier and also for variable limitation and to avoid data complexity. After the parameters have been set, the signal generator is turned on via checking the checkbox “*RF Output On*” on the HMC-T2200 GUI, and the time is recorded. Upon the completion of the frequency sweep, the recording is ceased. This procedure is performed three times in different sessions.

The output data comprises of three main parameters, including the timestamp in the YYYY-MM-DD.hh:mm:ss format, and the average x- and y- coordinates in arbitrary units (a.u.). The output data in.txt is transferred into an Excel format worksheet (.xlsx). Additional columns are added for conversion of the timestamps into seconds starting from 0, as the standard temporal reference, and the corresponding frequency based on the recorded time of turning on the RF output. The additional columns added and the headings are highlighted in the Excel worksheet as indication of the addition in the dataset. However, the original timestamp column is maintained for reference and data integrity. For the periodic sweep test, the system is left into autonomous data collection mode, where recording of the data may extend beyond the frequency sweep. The excess data output is truncated corresponding to the maximum frequency output at 20 GHz.

## III. Results & discussion

Four data collection sessions were conducted as per the methodology outlined in Section II, totaling four mosquito collection sessions and approximately 800 mosquitoes were used for all tests. The first is the null test while all hardware is disconnected from any power source. The following three tests comprises of mainly the periodic data collection. The following elaborates on the findings **(See** S1 Dataset).

### A. Null test

**Fig 2:**
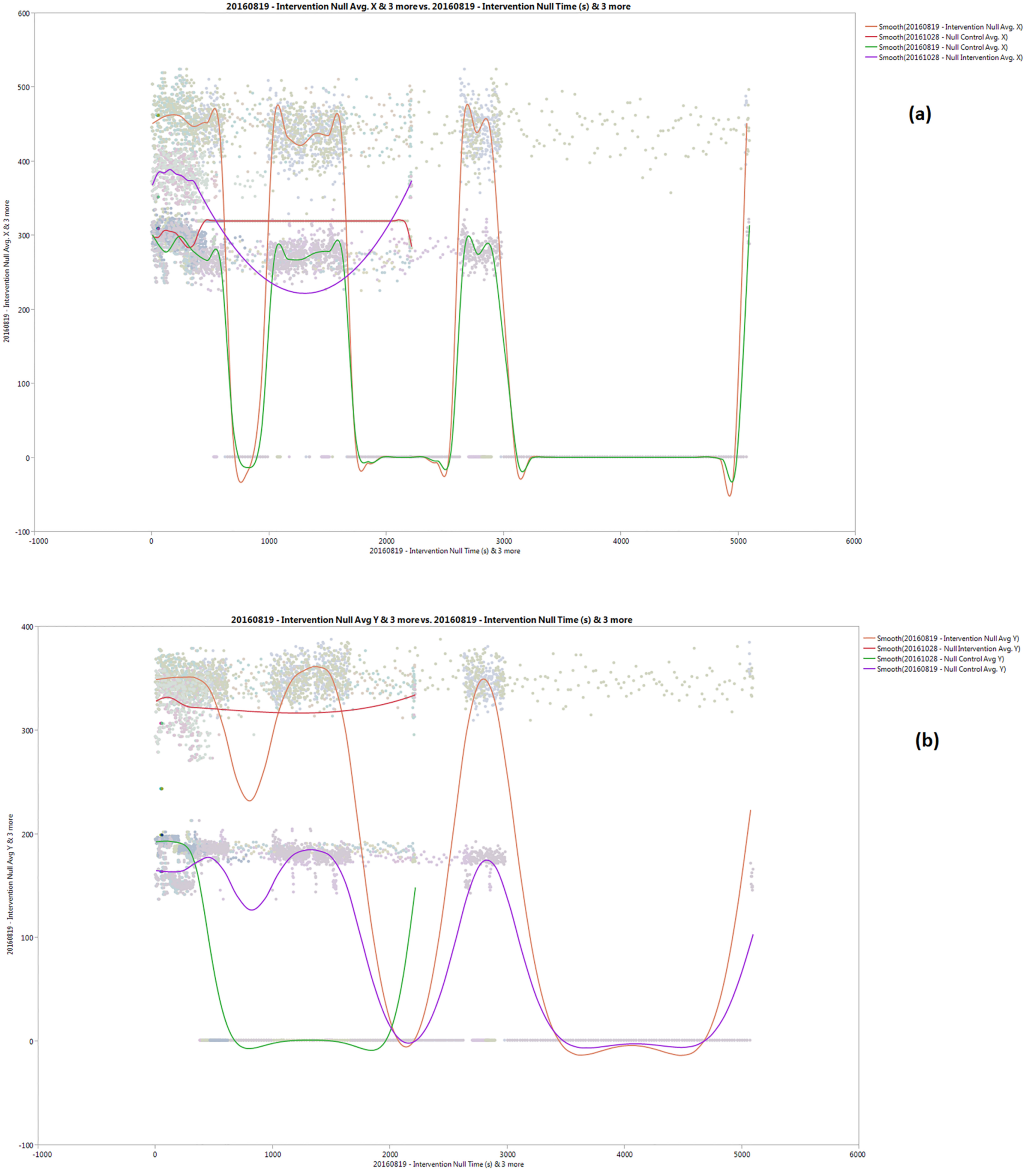
(a) Average Lateral Distance (a.u) and (b) Vertical Distance vs Time (s) for the null test.

In this test, the mosquitoes are released before turning on the apparatus for behavioral patterns to be observed. Upon the data collection, a graphical visualization using a statistical software (JMP Pro 12, SAS Institute Inc., 2015), of the average mosquito distance in arbitrary units (a.u.) as per defined by the video frame size, versus time in seconds. For this test, Fig 2 shows the average horizontal and vertical distance of the mosquitoes in arbitrary units (a.u.) from the rightmost side of the raster scan within the chamber captured from both intervention and control regions against time (seconds) for four cases:

From Fig 2(a) and 2(b), there are four series in the graph which shows the smoothed pattern of aggregated lateral and vertical distance from the left side of the chamber. Two of the series, where purple and red color in 2(a) and green and red in 2(b) show up to 36 minutes of behavior, and the remaining show up to 86 minutes. This comparison demonstrates the relative difference of the activities and predictably, the collectively random activity of the mosquitoes. Both control and intervention regions show non-uniform sporadic activities, most likely due to the fluctuations of the environment i.e. presence of human heat and temporal activity. As per visual observation, the mosquitoes have a high tendency to fly around during the initial period upon release into the chamber, before ultimately exhibiting dormancy.

During the periodic tests, three sessions of data logging were conducted, totaling approximately 33 hours of data collection period, where each session lasted up to 11 hours, covering mostly the scotophase period for the mosquitoes, as also performed in a previous study although the test was performed within a shorter period [25]. In this section, the aggregated spatiotemporal position is represented in smoothed plots, with the controls with each test for comparison. The output data was analyzed similar to the method in the previous section.

#### Periodic test 1: 01 September 2016

**Fig 3:**
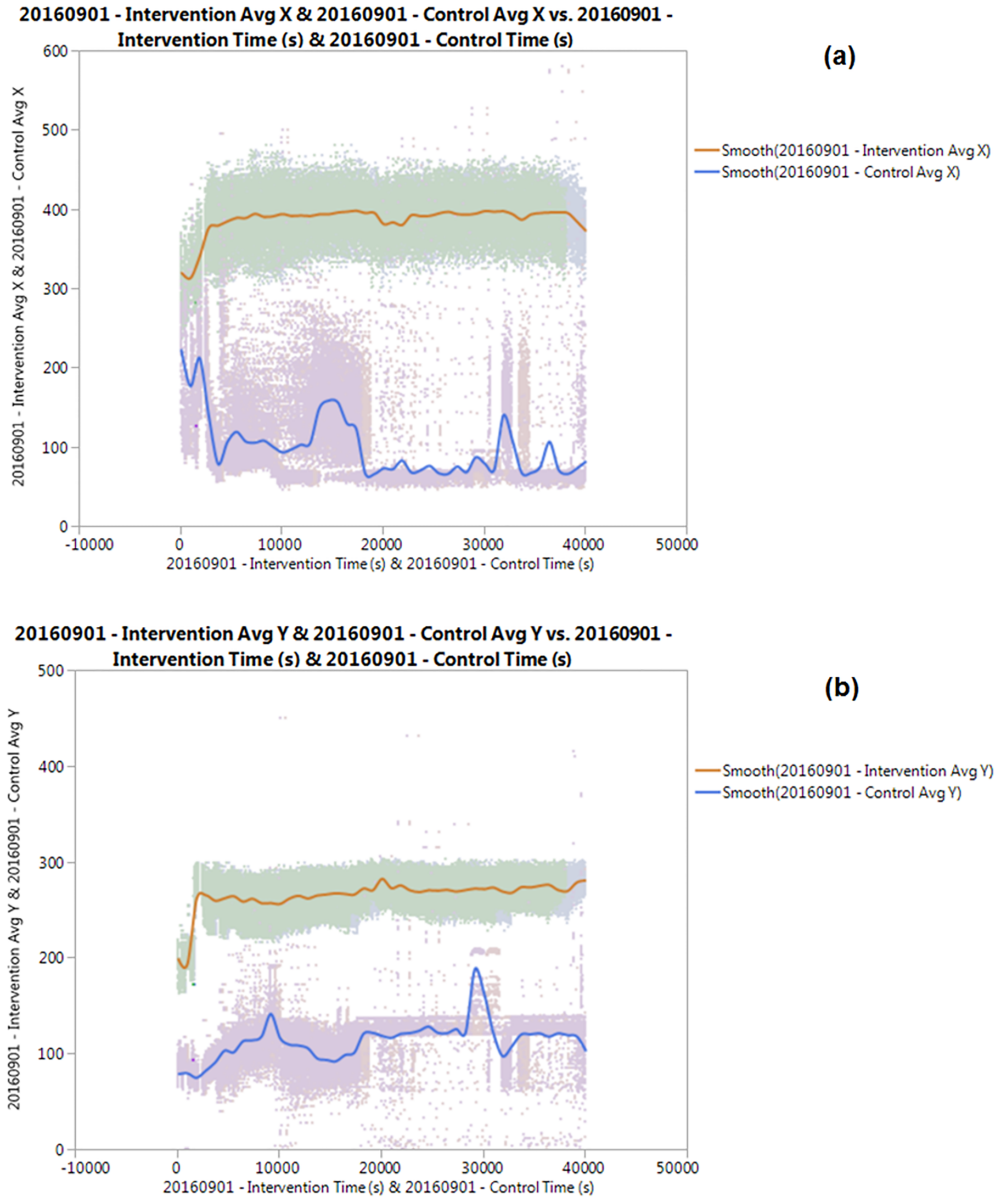
(a) Average Lateral Position (a.u) and (b) Vertical Distance vs Time (s) for the Periodic Test for intervention and control regions conducted on 01 September 2016.

A smoothed plot comparing the aggregated lateral and vertical positions of the mosquitoes versus time on 1^st^ September 2016 is shown in Fig 3(a) and 3(b):

Based on Fig 3(a), there is relatively more activity within the testing period in the Control region as shown from the blue smoother line, where during the first two hours indicate a more centred positioning of the mosquitoes. This excludes the ones resting outside the region of the camera view. After initial positions are assumed, from an initial position of 200 units, the readings indicated a left shift by approximately 100 units until the first hour of the test, followed by moderately constant fluctuations until the 4^th^ hour. This was followed a shift toward the left again for an hour for another approximately 100 units until the 5^th^ hour. This was followed by a relative monotony with less than 20% shift from the 5^th^ hour. An approximately 80-unit shift was observed toward the right after approaching the 8^th^ hour within the chamber until the end of the data collection. However, the orange-coloured series indicating activity in the intervention is significantly less observable, while only registering a slight change from the assumed positioning from the nearly monotonous spectrum during around the 5^th^ and 9^th^ hour toward the left of the chamber. This was probably due to very little mosquito activity, and also some might have escaped the detection algorithm due to occlusion, as also experienced in other studies where identification of mosquitoes may be lost after 10-20s [27]. Based on our observation, the mosquitoes tend to exhibit dormancy most of the time, resting on the edges of the chamber.

The vertical positions detected in the control region in Fig 3(b), however, shows increase during the 3^rd^ hour up to about 50 units and fluctuates throughout the session until peaking sharply during the 8^th^ hour up to around 180 units. The increase of activity during the end of the data collection may be attributed to the feeding behaviour as observed in female *Aedes Aegypti* in previous studies, which occurs in the early morning. As for the intervention region, however, shows very little vertical activity, except for a short period of movement towards the upper part of the chamber during the 5^th^ hour. However, the activity of the mosquitoes within this region suggests a despondency towards the stream of RF waves through the region, as can be observed from both lateral and vertical components.

#### Periodic test 2: 05 October 2016

**Fig 4:**
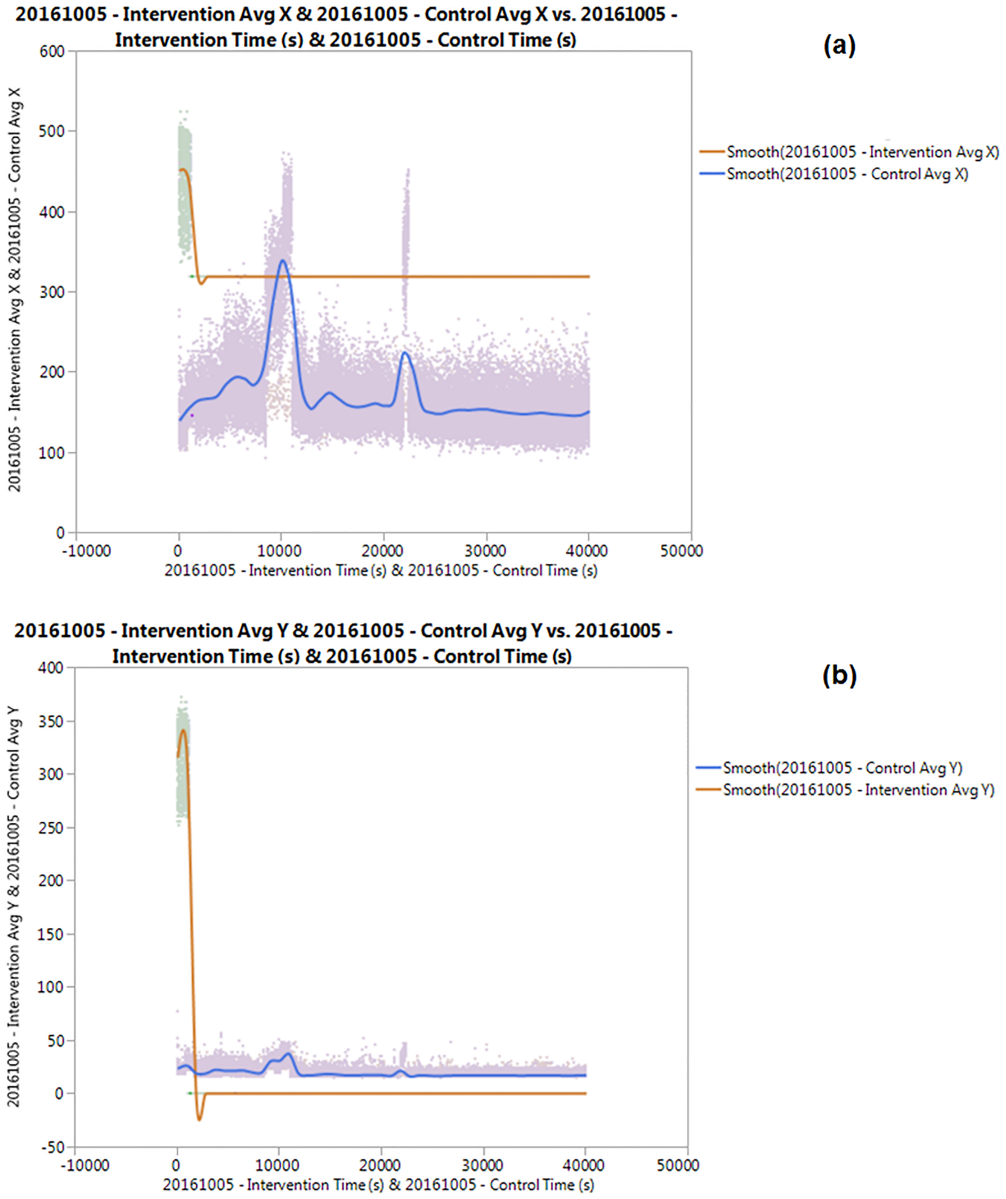
(a) Average Lateral Position (a.u) and (b) Vertical Distance vs Time (s) for the Periodic Test for intervention and control regions conducted on 05 October 2016.

Smoothed plots comparing the aggregated lateral and vertical positions of the mosquitoes versus time on 5^th^ October 2016 is shown in Fig 4(a) and 4(b):

Based on Fig 4(a), similarly convoluted patterns in the control region as shown by the blue line can be observed compared to the control region patterns in the previous Periodic Test 1. A rise in the lateral component occurred in relatively similar timing, which shows more leftward motion during the 4^th^ and 7^th^ hour and stagnating until the end of the test. However, the intervention region shows much less activity after the first hour, and stagnating during most of the period.

**Fig 5:**
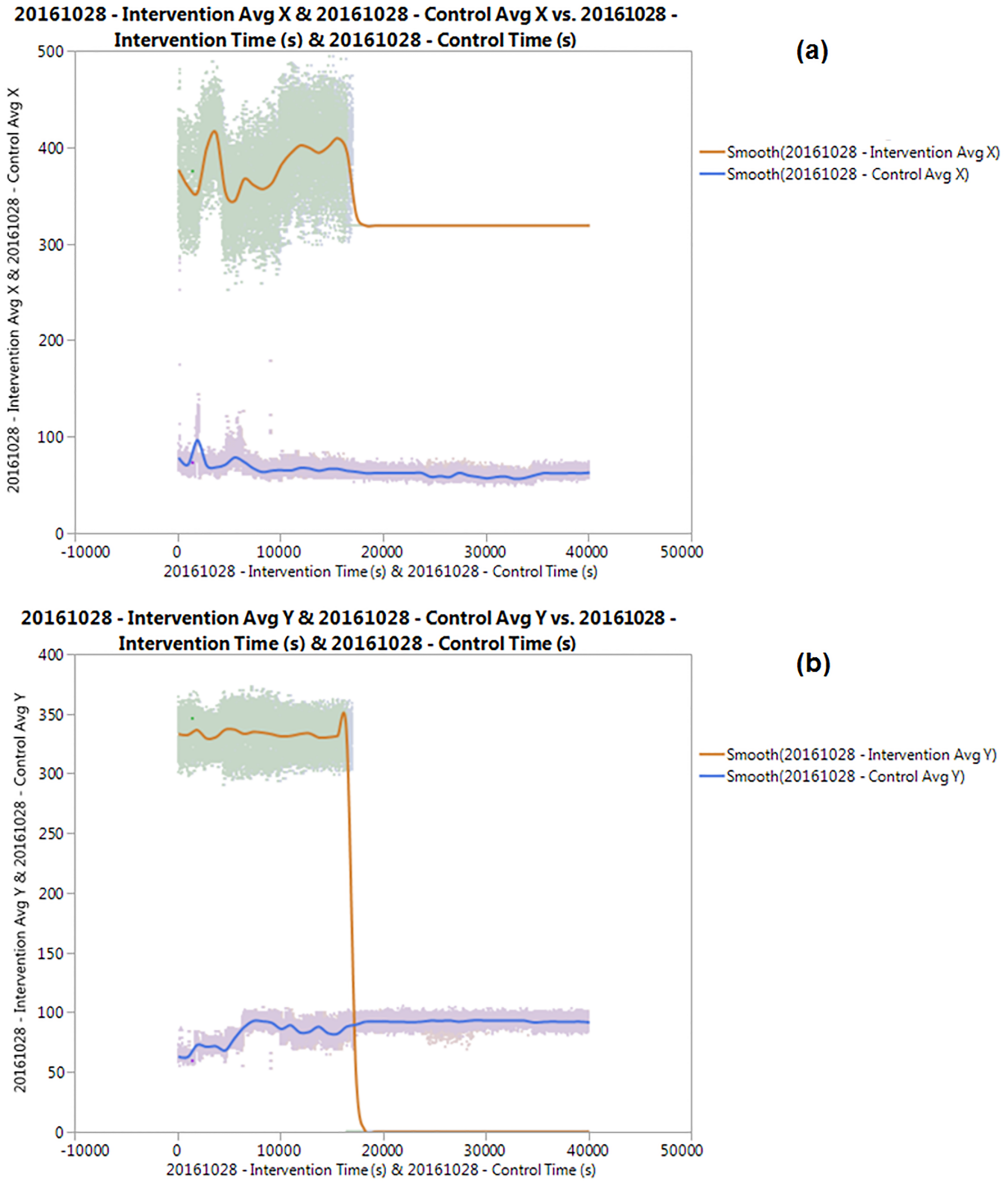
(a) Average Lateral Position (a.u) and (b) Vertical Distance vs Time (s) for the Periodic Test for intervention and control regions conducted on 28 October 2016.

The vertical motion for the control region as shown in Fig 5(b), however, shows much less significant change except a slight shift to the bottom of the chamber during the 3^rd^ hour. The intervention region shows a similar sharp decrease in the vertical motion compared to the lateral component, most probably due to most of the mosquitoes resting on the edges of the chamber and continues to show virtually no observable activity afterward. Again, this may be due to occlusion and detection issues. However, our estimation also inclines toward the non-activity of the mosquitoes against the RF streams, which is prevalent in *Anopheles gambiae* mosquitoes where they tend to preserve energy when no stimuli is present [27].

#### Periodic test 3: 28^th^ October 2016

Smoothed plots comparing the aggregated lateral and vertical positions of the mosquitoes versus time on 5^th^ October 2016 is shown in Fig 5(a) and 5(b):

Fig 5(a) showed the relatively stagnant lateral activity of the mosquitoes in the control region throughout the testing period, except a slight movement toward the right side of the chamber region during the 1^st^ and 2^nd^ hour of the period for approximately 20 units. However, the intervention region shows more convolution during the first half of the testing period. This can be seen from the rise of the lateral motion towards the right side of the chamber during the 2^nd^ hour and slowly moving back left during the 3^rd^ hour. Consequently, the movement spectrum shifted to the right side again until the 5^th^ hour for around 50 units to the base of the line before fully stagnating until the end of the testing period. Again, this can be attributed to the mosquitoes moving out of the frame of observation to the edges of the chamber, as can be observed from Periodic Test 2.

The vertical components as shown in Fig 5(b) also showed similar patterns for the control region compared to its lateral counterpart with only about 30 units of maximum displacement, which suggests the mosquitoes were mostly in resting mode throughout the test. Part of the mosquitoes rested on the chamber panel within the observation raster, and the majority of others were resting on the edges of the chamber. The vertical component for the intervention region, however, shows a sharp drop from a nearly stable position at the lower part of the chamber edges of the observation from the start until around the 4^th^ hour, with a drop of around 330 units, indicating a sharp leftward shift in the 5^th^ hour. This indicates most of the mosquitoes chose to reside on the edges until the end of the experiment. Another explanation is due to the occlusion and lighting drift, which may also play a role in the absence of detection, which might be related the challenges in maintaining mosquito detection which was lost after some time in another study on *Anopheles gambiae* mosquitoes [27].

#### Collective positions versus frequency

The following figures show the collective positions of the mosquitoes versus frequency by compiling all three tests mentioned in all periodic tests into one. Since the frequency is a time-dependent function in this test, the pattern of the spectrum versus frequency is similar to the one versus time.

**Fig 6:**
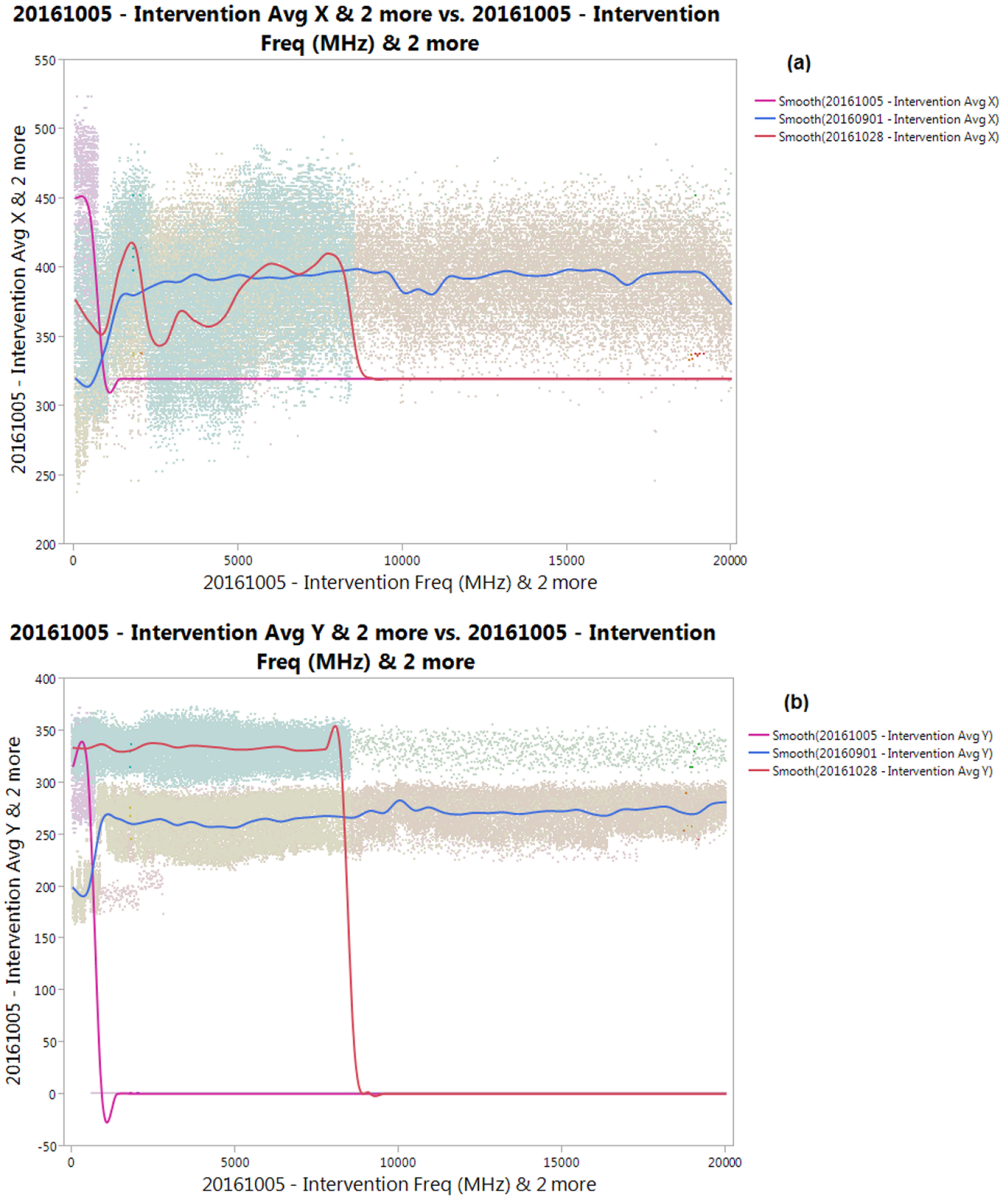
Collective (a) Average Lateral Distance (a.u) and (b) Vertical Distance vs Frequency (s) for the Periodic Test.

Based on Fig 6(a), by analyzing the pattern changes in the plots, it can be seen that there is little consistency in the flight patterns of the mosquitoes against all the frequencies for the three tests. This can be manually observed by examining the changes in the pattern, rather than the plot per se. Both lateral and vertical flights have very different reactions in the pattern changes. Fig 6(a) shows the plot on 28^th^ October 2016 (red line), where the changes of the direction toward the right side of the chamber occurred between 880.00 until 1830.00 MHz (observation provided via JMP Pro 12 Graph Builder), which shows a furthering of the mosquitoes from the source of the RF stream. This was followed by a significant 65 unit distance shift towards the RF source until 2240 MHz. This is followed by another slight movement back against the antenna 4730.00 MHz. After another slight movement towards the antenna on the left, the spectrum shifted towards the right again until 8937.00 MHz. This is again followed by a left shift until 10101.00 MHz. However, this shift only occurred within <10 units, which is less than 2% of the observation raster scan. Another shift towards the right side of the chamber occurred until 11484.00 MHz for 14 units and sharply drops toward the left until the end of the test period.

For the test occurring 1^st^ of September 2016 (blue line), the results are much less convoluted. The captured positions shows a sharp rise between 440.00–1267.00 MHz of 105 units. However, following this sharp rise, there apparently is negligible change throughout the spectrum except a slight dip toward the left between 9610.00–11330.00 MHz for less than 2% of the region of observation. The pattern shown by the test conducted on 5^th^ of October 2016 (purple line) shows a much less reaction from the test subjects, with only an initial movement towards the antenna during the first part between 150.00–1060.00 MHz for 162 units followed by the constant readings until the end of the test. These constant readings are, in our opinion, due to the inactivity of the mosquitoes which most frequently reside on the foam edges while occasionally moving.

Fig 6(b) shows the collective vertical flight patterns versus frequency, where both tests on 28 Oct 2016 and 01 Sept 2016 show similar patterns from 1170.00–7910.00 MHz, except the latter has an initial downward path in the observation from 0.00–1157.00 MHz for 78 units, whereas the test conducted on 5^th^ Oct 2016 (purple line) showed a sharp drop from within the same spectrum range for 340 units. The flight patterns of all three series stayed constant without any significant changes throughout the frequency spectrum. Any constant readings are most likely due to the presence of noise in the matrix of the processed frame, however this also demonstrates the relevancy of analyzing the pattern using this method due to the necessity for a significant number of mosquitoes needed to overcome the noise spectrum. Therefore, the viability of this technique to assess the collective behavior of the mosquitoes in this paper is maintained.

According to previous studies on investigating mosquito behavior, it is likely that the resting behavior, observed in most of the period during the test, is non-genetic trait, as observed on *Anopheles arabiensis* mosquitoes when subjected to a genomic sequencing test for comparing human/cow attraction factor [28]. Another factor contributing to convoluted activity is the presence of ambient carbon dioxide (CO_2_) as observed in *Anopheles gambiae* mosquitoes, which is probably the reason of the sporadic appearance in the spectrum as per the permeating CO_2_ in the lab environment [29]. Another study on *Anopheles stephensi* mosquitoes showed that mosquito fitness decreased if blood meals were restricted or delayed, as per the mosquitoes were not provided with any blood meals in this study [30]. From this exercise, it can be safely concluded that the mosquitoes exhibit a non-uniform behavior in the chamber. A more prevalent form of behavior observed from the data is dormancy, perhaps due to the mosquitoes’ adapting to the environment, as also reported in a previous study on the study of CO_2_ plumes toward mosquitoes, where mosquitoes tend to conserve energy with the absence of human odor [29].

Comparing the findings in this study with the current literature, researchers suspected frequencies emitted by between 27.00–401 MHz may induce oxidative effects and navigational capabilities towards different animals, however none was reported for mosquitoes [9]. However, these frequencies as mentioned are not tested in-lab with more precision, therefore subject to further investigation. Frequencies emitted by telecommunication towers between 800 to 2600 MHz have been known to disrupt a few insect species, including fruit flies, honey bees and cockroaches [8], although also none reported for mosquitoes.

## IV. Conclusions

The findings from this study show that the frequencies between 0.01–20 GHz exhibit inconclusive effects, which was proven from the data-based approach of examining the mosquitoes’ positions. To the authors’ estimation, after examining the data and comparing the output with the control data, there is negligible consistency in the reaction of the mosquitoes towards the whole spectrum of RF waves used in this study. In future, other researchers may adopt this method by examining other frequencies beside the ones tested in this study, and delve into in-depth investigations in this area for confirmation. It is hoped that further use of the algorithms in the software development in this study undergo more improvement for future entomologists to use.

In conclusion, this study has managed to document the methods of in-depth investigation of effects of low-powered RF waves towards mosquitoes. The materials include a low-cost and technically simple chamber with three sections, namely the antenna compartment, an intervention and control region with openings for mosquito release and the use of two low-cost USB-connected cameras attached to tripods, one camera for each region of observation. The mosquitoes are handled using mouth aspirators for transfer-to-chamber purposes.

As for the quantification of the average position of the mosquitoes, an open-source software coding using OpenCV and Visual Studio 2013 (S1 Source code and files and S1 Video for software demonstration) is demonstrated using the same tools in a previous related research on studying effects of human odor on the mosquitoes, with a light data size by generating text files for the data output (**See** S1 Dataset). The RF waves are emitted at close proximity toward the intervention region for analysing the effects of different frequencies against the mosquitoes. The frequencies are adjusted using another free software HMC-T2200 to operate a function generator HMC-T2100, Hittite Microwave Corporation^™^. The data output is plotted and analyzed using the statistical software JMP Pro 12, SAS Institute Inc., 2015.

## Supporting information

S1 Source code and files The source code file is a minor updated version from a previous publication in C++ language in Visual Studio 2013 environment with OpenCV v3.1, with a text file containing the code itself for direct access. (RAR)

S1 Video This video shows how the GUI development from the source code is initialized. (AVI)

S1 Dataset This dataset is an Excel File containing all test data from Null Test, Periodic Test 1, 2 and 3 for both Control and Intervention regions. Added columns are highlighted corresponding to the time in seconds and the frequency, which is a function of the time in seconds.
(XLSX)
